# Getting family planning and population back on track

**DOI:** 10.9745/GHSP-D-14-00012

**Published:** 2014-05-13

**Authors:** Malcolm Potts

**Affiliations:** a University of California, Berkeley, School of Public Health, Berkeley, CA, USA

## Abstract

After a generation of partial neglect, renewed attention is being paid to population and voluntary family planning. Realistic access to family planning is a prerequisite for women's autonomy. For the individual, family, society, and our fragile planet, family planning has great power.

For policy makers and for practitioners, the reward and satisfaction of family planning is that it is an inextricable mixture of helping individuals achieve their reproductive goals while also maintaining an awareness of the multiple ways in which demography has determined our past and will inevitably shape our future. Voluntary family planning programs since the 1960s have helped 48% of the world's population achieve replacement-level fertility or below.^1^ (Replacement-level fertility is the fertility rate at which each generation has only enough children to replace itself, and thus the population eventually stops growing. This is generally when the total fertility rate [TFR] is about 2.1 children per woman, although it can be at higher levels in countries with high mortality rates.^2^)

Without this reduction in family size, the number of people living on less than US$1.00 a day would not have been halved,^3^ improvements in education would have been slower (such as was shown in Thailand^4^), and there would not have been such a rapid decline in infant and maternal mortality. In many Asian countries, the rapid change in population structure from the introduction of voluntary family planning led to a “demographic dividend,” which helped lift millions of people out of poverty.^5^ The demographic dividend is the rapid economic growth that may result when a country transitions from high to low birth and death rates and the subsequent change in the age structure of the population—the smaller young dependent population with a larger working-age population translates into fewer people to support.^6^

For a variety of reasons, which will be touched on shortly, following the 1994 United Nations (UN) International Conference on Population and Development (ICPD) in Cairo, family planning budgets fell.^7^ The fertility decline that had begun in some high-fertility countries, such as Kenya,^8^ stalled. The policy community paid less and less attention to population as a factor in development, in resilience to climate change and to the long-term sustainability of the global economy. The urgent need for new family planning initiatives, particularly in sub-Saharan Africa, was set aside. Today, there are approximately 424 million African children aged 14 or under. In 2050, Africa could have 770 million children, allowing a great deal of demographic momentum to build up.^1^ It is questionable whether some economies in sub-Saharan Africa will be able to benefit from the demographic dividend in the way that much of Asia did.^9^ These were costly mistakes that will help shape the remainder of the 21st century.

After providing an overview of global population projections and describing exactly what is at stake, this article reviews the evolution of the population and development debate and the resultant family planning policies from the 1960s to present day. In particular, it argues that while ICPD was a critical milestone in the history of population and development as well as in the history of women's rights, it was also a turning point at which the focus was unfortunately taken off of voluntary family planning, interrupting an earlier decline in fertility.

## BIG NUMBERS

Global population projections to 2100 from the UN Population Division present some profoundly different scenarios (Figure). The high and low population variants differ by half a child above or below the medium variant. Virtually all biologists and climatologists, along with an increasing number of sensible economists, would agree that a world with 6.8 billion people in the year 2100 (low-variant projection) would be more likely to be biologically sustainable, healthier, more educated, and less violent than one with 16.6 billion (high-variant projection).

**FIGURE.**
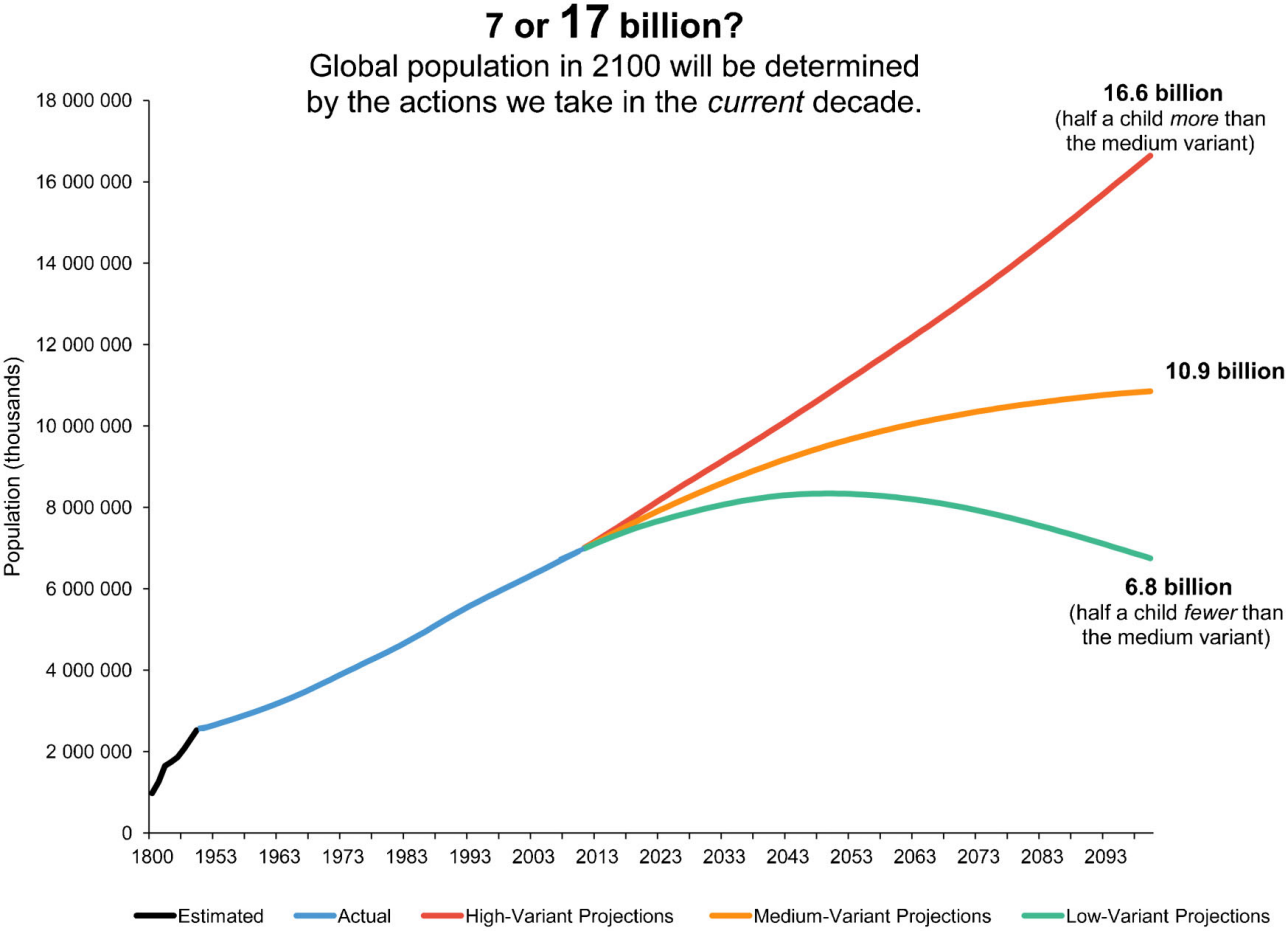
Population Estimates and Projections, 1800–2100 Data from the United Nations.^1^

Nearly all global population growth in the future will be in the less developed countries.^1^ Those countries can be divided further into 2 groups: countries such as Bangladesh and Kenya that have a reasonable chance of achieving replacement-level fertility in the foreseeable future, and those countries that still have a high birth rate. Since the UN first identified 25 least developed countries in 1971,^10^ only 1 country with a population of over 1 million (Botswana) has graduated to the “developing” country category^11^ (and the list included 48 countries as of June 2013). The least developed countries often have corrupt governments; the status of women is commonly abysmal in these countries; and rapid population growth in them is typically associated with a large number of poorly educated young men with few job opportunities—a recipe for violence and conflict.^12^ Currently, the least developed countries together have about 898 million people.^1^ In 2100, the medium UN population projection for these least developed countries is 2.9 billion, or as many as there were people in the whole world in 1960. The high estimate is 4.3 billion, or equivalent to the size of the world's total population in 1980.^1^

Virtually all the world's population growth in the future will be in less developed countries.

Each of the 3 projections in the Figure is possible. Which one the world follows will shape the world our children and grandchildren inherit and will play an immense role in determining global poverty, hunger, gender equality, and environmental sustainability outcomes. Thinking in terms of half a child may seem a bit laughable, but it emphasizes that the future well-being of the planet turns on a relatively small change in average family size.

Considerations of population and family planning have been pushed off the policy agenda for many reasons, among which are religious and political conservatism, donor fatigue, and competition with funding for HIV/AIDS. Some economists assert that family planning programs are unnecessary because if couples want fewer children, the free market will solve their problems. Unfortunately, family planning is not a free market but one beset with biases and barriers that are not based on evidence. Population and family planning were regarded as too controversial to include as targets in the first round of the Millennium Development Goals, and they continue to be excluded from many discussions of climate change, conservation, and food security. For example, the 2013 annual Water for Food Conference, attended by more than 450 people from 24 counties and supported by the Bill & Melinda Gates Foundation, provided no space to discuss how family planning might alter the demand side for food.^13^

Population and family planning also have been pushed off the agenda due to sheer enormity of the numbers. It demands an almost impossible intellectual and emotional effort to try and visualize what 16.6 billion means—counting 1 person per second would take 33 years to register just 1 billion people. In Paul Slovic's powerful phrase, we are “numbed by numbers.” Slovic, an experimental psychologist, asked a sample of Americans how much money they would donate to help an individual hungry child in Africa. He identified such a child with a name, a photograph, and brief biography. The average donation offered was US$2.00. However, when Slovic told exactly the same story, but added that there were a million other hungry children, people donated *less*. When statistics were presented without a human face, people gave the *least*. “Grim statistics,” Slovic observes, “themselves paralyze us into inaction.”^14^

Unless you are totally numbed by numbers, then the UN population projections are staggering predictions. Suddenly a half-child more or half-child less becomes of existential importance. Does voluntary family planning offer a proven way to ensure that half-child difference?

## MODELS OF FERTILITY DECLINE

It has long been recognized that rapid population growth can hold back socioeconomic development, but policy makers remain divided on the weight to be given to family planning programs.^15^^–^^17^ For a long time, family planning policies have been split between those who hold that “Development is the best contraceptive,” and those who think that “Contraception is the best development.” These 2 polar opposites have deep roots.

Two models of fertility decline have divided policy makers: “Development is the best contraceptive” vs. “Contraception is the best development.”

When the U.S. Agency for International Development (USAID) first received support for international family planning in the 1960s, many demographers and economists were wedded to the standard “development is the best contraceptive” explanation of the demographic transition. Based on the history of Europe and North America, it was held that as deaths rates fell and as income and education improved, birth rates always fell. Couples, it was argued, made a rational decision to have fewer children. Socioeconomic change, it was asserted, was both necessary and sufficient for fertility decline: family size would fall “with great effectiveness” without the “assistance of modern contraceptive techniques.”^18^ The first family planning programs that USAID supported were dismissed as “quackery” and “wishful thinking.”^19^

The alternative perspective was captured in a statement by Reimert Ravenholt, physician and epidemiologist and the first director of USAID's population programs in the 1960s:

*It seems reasonable to believe that when millions of women throughout the world need only reproduce when they choose, then the many intense family and social problems generated by unplanned, unwanted and poorly cared for children will be greatly ameliorated and the now acute problems of too rapid population will be reduced to manageable proportions.*^20^

This pragmatic approach emphasized access to modern contraception and the need to remove the many non-evidence-based barriers to family planning.^21^^–^^23^ When contraception and abortion were illegal in the United States, it took 58 years (1842 to 1900) for the TFR to fall from 6 to 3.5 children per woman.^24^ When modern family planning was available in Thailand, a similar transition occurred in a mere 8 years (1969 to 1977). In Bangladesh, and to some extent in Brazil, Indonesia, and other countries, realistic access to family planning brought the birth rate down ahead of major socioeconomic improvements. The same thing has happened in Addis Ababa, Ethiopia, with a TFR of 1.5, which is unique among African capital cities.^25^ We associate the Islamic Republic of Iran with conservative ways, but once women had better access to family planning, the TFR actually fell more rapidly than in China—and without a one-child policy.^26^ Interestingly it needs to be recognized that the TFR in China had fallen from 6.5 to 2.5 as the result of largely voluntary family planning, even before the one-child policy was introduced.

Although the standard model of the demographic transition has come under increasing academic criticism,^27^^–^^29^ it remains influential in many policy environments.^30^ Only last year, Jamison et al. wrote, “Families choose to have fewer children when they realize that the mortality environment has changed.”^31^ However, heterosexual couples have intercourse hundreds of times more frequently than is necessary to conceive even a large family.^32^^,^^33^ Therefore, the default position for couples is a large family. No woman can “choose” to have fewer children until she is given the means and information to separate sex from childbearing. In Niger, the “mortality environment” has changed dramatically (between 1990 and 2012, the infant mortality rate fell from 137/1,000 live births to 63/1,000), but the TFR remains at 7.6—the highest in the world.^34^

No woman can choose to have fewer children until she has the means and information to separate sex from childbearing.

Child marriage is a human rights abuse, mainly concentrated in the least developed countries. Rolling back the age of marriage is also a demographic imperative. Where child marriage is widespread, women will never be able to manage childbearing unless the age of the first birth is radically increased.^35^ Investing in girls and young women is a non-negotiable part of any strategy to slow population growth and to enable the least developed counties to move forward. Pilot studies in Northern Nigeria show it is possible to keep 70% to 80% of girls in secondary school in a region where only 4% of girls previously entered secondary school and none had completed their secondary education (personal communication with Daniel Perlman, Research Medical Anthropologist, University of California, Berkeley, Bixby Center for Population, Health & Sustainability, and Co-Director, Bixby/Fogarty International Center Population and Health Program, Nigeria, 2013). Bringing such programs to scale could require billions of dollars a year. Reinserting the population factor where it belongs in the family planning equation will give us the confidence and the evidence base to think big.

Investing in girls' education is a critical part of any strategy to slow population growth.

## TRAGIC EPISODES OF COERCION

Paradoxically, if development really is the best contraceptive, then we have to ask: What will happen in a situation where rapid population growth is undermining socioeconomic progress? Indian Prime Minister Indira Gandhi faced this conundrum in the late 1970s. Under her leadership, in 1976 Dr. Karan Singh, then Minister for Health and Family Planning, released the following statement^36^:

It is clear that simply to wait for education and economic development to bring about a drop in fertility is not a practical solution. The very increase in population makes economic development slow and more difficult of [an] achievement. The time factor is pressing and the population so formidable, that we have to get out of this vicious circle through direct assault upon this problem … Where [an Indian] state legislature, in the exercise of its own powers, decides that the time is right and it is necessary to pass legislation for compulsory sterilization, it may do so.

The coercive family planning measures that Indira Gandhi introduced were among the unacceptable episodes of forceful family planning in our modern history. This step lost her the next election. It seems unlikely, however, that she would have gone out of her way to introduce unpopular policies if she had believed that development was the only way to lower the birth rate. What she did not understand was that if you respect women and remove the unjustified barriers to family panning then family size would fall, even without significant socioeconomic progress. Had she known that, then she might not have felt compelled to introduce coercive policies.

## THE ICPD: REALITY AND MYTH

The Programme of Action adopted at the ICPD balanced a realistic concern for confronting rapid population growth with an eloquent statement of voluntary family planning as a human right^37^:

Principle 4: Advancing gender equality and equity and the empowerment of women, and the elimination of all kinds of violence against women, and ensuring women's ability to control their own fertility, are cornerstones of population and development-related programmes.

Principle 5: Population-related goals and policies are integral parts of cultural, economic and social development, the principle aim of which is to improve the quality of life of all people.

However, a different perspective was widely promoted after ICPD. Hodgson and Watkins describe how^38^:

… a vision of fertility decline as a necessary consequence, not a cause, of large societal changes was to provide the frame that feminists would modify for later use at the 1994 Cairo conference.

Poignantly, such commentators were espousing the very paradigm that drove Gandhi to implement the most loathsome abuses in the history of family planning.

Another group of advocates following the ICPD wanted to push population off the table in order to secure what they believed would be greater funding for the broader goals of women's empowerment.^39^ The late Joan Dunlop, a powerful leader in the group, explained in an interview with author Michelle Goldberg^40^:

What we wanted to do was, rather, simply [not]* *throw the baby out with the bathwater; we wanted to redirect the money. We knew there were huge streams of money going into contraceptive development, and we wanted that money to go in a different direction.*

The baby was indeed thrown out with the bath water. Dunlop's desire to divert the “huge streams of money going into contraceptive development” was a classic case of being numbed by numbers. These “huge streams of money” that Joan Dunlop dreamed of diverting to the needs of women had never exceeded $15 million in any one year prior to ICPD.^41^ The goal of helping women needed a budget of billions of dollars.

The strategy to “move the money in a different direction” benefited from framing everything that happened in international family planning prior to ICPD as intrinsically coercive. Dunlop and others began a viral myth that is encapsulated in a book called *Fatal Misconceptions* by Matthew Connelly. Connelly engages in a systematic attempt to rewrite history to fit the ideology that all family planning programs are coercive.^42^ However, even Connelly was unable to verbally tar and feather Ravenholt, the charismatic advocate of voluntary family planning mentioned earlier^43^:

Ravenholt's office was virtually alone in its policy of refusing support for programs to create demand for contraception. He argued that supplying “unmet need” would be enough to solve the problem of population growth, or was at least worth trying before trying anything else. Many of his superiors and subordinates disagreed, and pressed Ravenholt for experiments with incentives.

Ravenholt continued to insist that incentives were not only unnecessary but also inappropriate.

## GETTING BACK ON TRACK

We live in a complex, interconnected world facing unique problems that, if not tackled, could cause great pain,^44^ or even threaten a collapse of civilization as we know it.^45^ Can we create a world that lives within ecologically sustainable limits? Can we avoid ever-growing inequities between the least developed countries and the rest of the world? Can we create a more stable, less violent world? Family planning is only one factor in answering these existential questions, but family planning is a prerequisite for any solution.

Family planning is one essential solution, albeit not the only one, to solving some of the world's complex problems.

The Family Planning 2020 (FP2020) partnership, with the goal of reaching 120 million additional women with voluntary access to family planning within the next 6 years,^46^ is an important step toward a more balanced and evidence-based approach to family planning and population after a generation of relative inactivity.

Family planning saves maternal and infant lives. It has reduced maternal deaths by 40% in the past 20 years.^47^ A child conceived within 6 months of a prior birth is 60% more likely to die than a child whose conception was spaced by 2 years.^47^ Family planning can trigger economic development, and it can assist in both mitigation of and adaptation to climate change.^48^ Family planning is an investment that pays for itself in reduced health and educational costs, yet budgets are not commensurate to its impact.^49^ Slowing birth rates through voluntary family planning can preempt conflict and political instability.^50^ Family planning is listening to what women want, not telling them what to do. An estimated 47,000 deaths from unsafe abortion occur each year^51^—evidence written in blood that millions of women want fewer children but do not have realistic access to modern contraception.

In 1969, I was invited to deliver the Tenth Darwin Lecture in London. I ended by saying^52^:

Some writers are asking, “What is beyond family planning?” They are talking about incentives where previously they spoke of motivation. Reports of transistor radios are becoming tales of compulsory sterilization or hormones in the drinking water. I think this trend is dangerous and unnecessary. The ideal of voluntary parenthood is an exceptionally important freedom to preserve. I fear it is threatened with erosion because we are failing to make a free choice of contraceptive methods available.

Forty-five years later, I still believe this to be true. I also suggest that there is an urgent need to reunite a concern for individual freedom with an emphasis on the need and opportunity to slow rapid population growth in a human rights context.

It is time for those advocating improvements in the status of women to link arms with those deeply worried about a world that is exceeding the capacity of the biosphere to sustain human activity. The investments that need to be made, and the policies that need to be put in place, are identical for both groups: meet the unmet need for family planning and advance girls' education. Doing so will start the world population on a trajectory of 6.8 billion people by 2100. A world of 11 or 17 billion (Figure) could well find it impossible to make the transition to a biologically sustainable economy.

Voluntary family planning is founded on the core belief that every woman has the right to decide how to use her own body. It is the freedom that separates a slave from a free person. Making family planning readily accessible is the first step in breaking the shackles of reproductive slavery. It is also the first step in saving civilization from destroying itself. For the individual, the family, society, and our fragile planet, it is imperative to get voluntary family planning and a commitment to slowing rapid population growth back on the same track.

## Footnotes

* Author Goldberg recognizes a typo in her published text.
